# Data on the use of dietary supplements in Danish patients with type 1 and type 2 diabetes

**DOI:** 10.1016/j.dib.2018.11.144

**Published:** 2018-12-05

**Authors:** B. Ewers, E. Trolle, S.S. Jacobsen, D. Vististen, T.P. Almdal, T. Vilsbøll, J.M. Bruun

**Affiliations:** a Steno Diabetes Center Copenhagen, Gentofte, Denmark; b The National Food Institute, Technical University of Denmark, Soeborg, Denmark; c Rigshospitalet, Copenhagen, Denmark; d Department of Clinical Medicine, Faculty of Health and Medical Sciences, University of Copenhagen, Copenhagen, Denmark; e Department of Nutrition, Exercise and Sports, Faculty of Science, University of Copenhagen, Frederiksberg, Denmark; f Department of Medicine, Randers Regional Hospital, Denmark

## Abstract

The data in this article describe the use of dietary supplements in Danish patients with type 1 diabetes (T1D) and type 2 diabetes (T2D). The data were collected from a web-based dietary survey on dietary habits in 774 patients with T1D (*n* = 426) and T2D (*n* = 348). The data demonstrate that 99% of the patients with diabetes use dietary supplements with no gender differences. In comparison, only 64% in the general population use dietary supplements [2].

A higher proportion of people in the general population use multivitamin/mineral supplementation as compared to patients with diabetes (48% vs. 34–37%) and a higher proportion of women than men with diabetes use multivitamin/mineral supplementation (T1D: 43% women vs. 26% men and T2D: 45% women vs. 34% men). More patients with diabetes than the general population use supplements such as calcium together with vitamin D, vitamin D, vitamin B, vitamin C, vitamin E, magnesium, calcium, Q10, ginger, garlic, and other herbal supplements.

**Specifications table**

**Table:** 

Subject area	*Nutrition*
More specific subject area	*Dietary supplementation in adults with type 1 and type 2 diabetes*
Type of data	*Table and text file*
How data were acquired	*Web-based questionnaire*
Data format	*Raw, analyzed*
Experimental factors	*The criteria used for including patients in the study and how data were collected has been described in Ewers et al.*[1]
Experimental features	*Data were analyzed by using SPSS software for Windows, version 22.0 (IBM Corp, Armonk, NY, USA)*
Data source location	*Copenhagen area, Denmark*
Data accessibility	*The data are with this article*
Related research article	B. Ewers, E. Trolle, S.S. Jacobsen, D. Vististen, T.P. Almdal, T. Vilsbøll, et al. Dietary habits and adherence to dietary recommendations in patients with type 1 and type 2 diabetes compared with the general population in Denmark. Nutrition 2018 (in press) [1].

**Value of the data**

• The data presented in this article provide new information about the use of dietary supplementation in patients with type 1 and type 2 diabetes.
• The data provide important new evidence for differences in use of dietary supplementation between men and women.
• The data can be used to identify differences in the use of different dietary supplementation in patients with diabetes and the general population.
• The data can be used by clinicians and academia for further research and as reference.

## 1 Data

The data article presents data on the intake of dietary supplementation in adult Danish patients with T1D and T2D. In Table 1, the percentage of dietary supplementation among male and female patients with T1D and T2D are presented. The supplementations are divided into all dietary supplements, multivitamin/mineral supplements, fish oil, calcium together with vitamin D, vitamin D and other supplements including herbal supplements. The data are compared with previous reported data on intake of dietary supplements in the general Danish population [2] based on the Danish National Survey of Dietary Habits in Denmark 2003–2008 [3].

**Table 1:** Percentage use of dietary supplementation among patients with diabetes and the general population.

**Dietary supplementation**	**T1D** (*n* = 426)	**T2D** (*n* = 348)	**General population** (*n* = 3037)
	%	%	%
All dietary supplements,			
- Men	98.2	98.0	57.0
- Women	99.0*	100.0*	71.0
- All	98.6	98.6	64.0
Multivitamin/mineral,			
- Men	26.3	33.5	43.0
- Women	42.6*	44.6*	53.0
- All	34.0	37.0	48.0
Fish oil,			
- Men	20.3	21.8	18.0
- Women	27.3	24.8	25.0
- All	23.7	22.6	22.0
Calcium and vitamin D,			
- Men	11.1	10.9	5.2
- Women	30.1*	33.7*	17.9
- All	20.4	17.5	11.7
Vitamin D,			
- Men	19.8	27.0	6.4
- Women	25.4	43.6*	10.5
- All	22.5	31.8	8.5
Other supplements^a^,			
- Men	53.0	54.4	14.8
- Women	47.4*	37.6*	24.3
- All	50.2	49.6	19.8

a Other supplements including vitamin B, C, E, magnesium, zinc, calcium, Q10, garlic, ginger, other herbal supplements.

* Statistical difference (*p* < 0.05) between men and women with diabetes.

## 2 Experimental design, materials and methods

Data on use of dietary supplementation were collected in a cross-sectional dietary study of patients with T1D and T2D [1]. Data on the type of diabetes and gender were extracted from an electronic medical record in the outpatient clinic where the participants were recruited from. Participants were asked to report the use of all dietary supplements. Response to the use of the presented dietary supplements in Table 1 (yes/no) was mandatory. Furthermore, participants were asked to report the use of other dietary supplements (open-ended questions category). Data on the use of dietary supplements were compared between men and women with T1D and T2D by using the Chi-square test for differences in proportions. A two-sided significance level of *p* < 0.05 was used. All statistical analyses were performed with the SPSS software for Windows, version 22.0 (IBM Corp, Armonk, NY, USA).
