# Supplementary material for: Data on the use of dietary supplements in Danish patients with type 1 and type 2 diabetes
Source: Data Brief. 2018 Dec 5;22:241–4. doi: 10.1016/j.dib.2018.11.144 (PMC6305888; doi:10.1016/j.dib.2018.11.144)
Supplement: Supplementary file 1 — Supplementary material [file mmc1.docx]

Conflict of interest none.
